# Restricting the use of sunbeds to prevent skin cancer

**DOI:** 10.2471/BLT.17.021217

**Published:** 2017-12-01

## Abstract

Young people – especially women – are risking their health in pursuit of a tanned skin that they associate with celebrity, beauty and wellbeing. Tatum Anderson reports.

Australian Clare Oliver was just 26 when she died of skin cancer. Speaking from her hospital bed in the city of Melbourne to an Australian television crew, she blamed the spread of melanoma on her regular use of sunbeds.

“I’ve been told I have weeks to live and I don’t think a solarium, and looking good with a golden tan is worth that,” she said.

Ten years after her death in 2007, Oliver is still very much in Australians’ minds, says Craig Sinclair, head of the Prevention Division at the Cancer Council Victoria, a World Health Organization (WHO) Collaborating Centre for ultraviolet (UV) radiation.

Oliver’s interview triggered a media frenzy and a shift in the debate on sunbeds in Australia, prompting the state of Victoria to ban their use by people aged under 18 years. The move was quickly endorsed by the federal health minister and the prime minister of Australia at the time.

By 1 January 2016, all six Australian states had gone a step further by imposing a ban on the use of commercial sunbeds for everyone.

Australia is one of several countries to have placed restrictions on the use of sunbeds to reduce skin cancer.

The use of artificial tanning devices – including sunbeds, stand up booths and facial tanners – is responsible for an estimated 450 000 non-melanoma skin cancer cases and more than 10 000 melanoma cases each year in the United States of America, Europe and Australia combined. 

These figures do not account for eastern Europe and Latin America, where sunbeds are also commonly used.

Women – especially young women – are the biggest users of artificial tanning devices.

“Skin cancer incidence has risen among light-skinned populations and artificial tanning devices have contributed to this increase over the last three decades,” says Emilie van Deventer, team leader of WHO’s radiation programme in Geneva.

This year, WHO issued guidance on the various policy options for countries seeking to restrict solarium use. According to *Artificial tanning devices: public health interventions to manage sunbeds, *countries need to educate the public about the health risks of UV exposure from sunbeds and apply regulations to restrict their use. 

“There are several policy options for governments. They can make licensing for tanning salons mandatory,” van Deventer says. “They can require tanning salons to inform customers about the health risks and they can prohibit the marketing and promotion of sunbeds.”

The WHO guidance provides examples of these and other policy actions countries are already taking, such as introducing additional taxes for tanning sessions. “This is similar to other taxes or levies on tobacco, alcohol, sugar and fat that are now commonly used to drive down demand,” van Deventer says.

Public health campaigns are useful, as seen in Australia, Denmark and Italy, but voluntary codes have largely failed to protect the public, the guidance notes.

A compulsory code of practice in Brazil failed because of the challenges of verifying compliance, says Rafael Fernandes, a specialist in regulation and inspection at the Agência Nacional de Vigilância Sanitária (ANVISA), the country’s health regulatory agency. 

For example, operators were supposed to limit the strength and intensity of sunbed equipment. However, inspectors did not always have the technical knowledge to gather the required data. In addition, it was a challenge to determine whether clients were minors, whether sessions were time-limited and whether clients received advice from operators on the risks. 

In 2009, Brazil imposed a general ban on sunbeds, becoming the first country to do so. ANVISA’s decision to impose the ban was based on the International Agency for Research on Cancer’s (IARC) classification of UV-emitting tanning devices as carcinogenic a few months earlier and its conclusion that there is no safe limit for UV from such devices.

The 2009 Brazilian law prohibited trade in artificial tanning devices and their use unless prescribed by a doctor for health reasons. In addition, lawmakers are considering new legislation on the distribution of sunscreen by the public health system and by employers for their outdoor workers.

For Sinclair, the general ban on sunbeds was feasible as a policy option for Australia’s six state governments because it was supported by three elements: scientific evidence of the harm to people’s health, government support to help sunbed operators switch to other services and economic studies showing the benefits of such a ban in terms of reducing related health costs. 

In addition, the sunbed industry lobby in Australia was relatively small at the time the general ban was imposed, Sinclair says. In contrast, the sunbed lobby in Europe and North America is powerful and vocal in challenging government policy to restrict sunbed operators’ activities. 

A 2007 study by researchers from the Queensland Institute of Medical Research was particularly influential. They predicted that for a cohort of 100 000 young Australians, more than 1000 melanomas and 12 000 squamous cell carcinomas could be prevented by banning sunbeds, saving 12.2 million Australian dollars (US$ 9.4) in health-care expenditure. 

Sinclair says that it is partly thanks to intense public health messaging on the risks of UV exposure in Australia, which has one of the highest rates of skin cancer in the world, that the ban has been well received by the public.

“We have a 30-year history of sun protection campaigns starting with *slip, slop, slap,*” he says, referring to an acclaimed campaign in Australia and New Zealand urging people to “slip” on long-sleeved clothes, “slop” on sunscreen and “slap” on their hats.

The ban in Australia helped challenge widespread misperceptions. “Many people wrongly assumed that tanning on a sunbed was healthier than tanning in the sunshine. Some people thought that if the skin tans, but does not burn, then there’s no damage to their skin,” he says.

Since the general ban on sunbeds, more people in Australia read about melanoma– the most lethal type of skin cancer – on the internet and looked for alternatives, such as spray tans. Moreover, there is little evidence of a black market, when individuals buy sunbeds for personal use, which is not restricted, and provide illicit solarium services from their homes.

In Brazil, the national ban has also exploded myths about sunbeds and tanning. “Solarium equipment is very simple and only the lamps are visible, so I don’t think people realised how dangerous it is,” Fernandes says.

Several tanning salons in Brazil have brought lawsuits to lift the national ban and some of those are still ongoing today.

Perhaps Brazil’s general ban on the use of sunbeds was quick to gain public support because solariums were not as popular as they are in other – usually colder – countries in Europe and North America.

Fernandes says another benefit of a sunbed ban has been that more people are aware of the health risks of UV exposure.

Last year, echoing IARC’s findings, the Scientific Committee on Health, Environmental and Emerging Risks of the European Commission issued a scientific opinion that “there is no safe limit for exposure to UV radiation from sunbeds” and therefore restrictions on the use of artificial tanning devices are justified, including an outright ban on them. 

The committee also made it clear that the beneficial effects of sunbed use, such as for the generation of vitamin D, are outweighed by the adverse effects. “There is no need to use sunbeds to induce vitamin D production because alternative sources of vitamin D are readily available,” the committee said.

While the arguments for a general national ban are strong, however, only Brazil and Australia have taken this policy path so far. Countries where sunbeds are popular, such as Germany, have pursued partial restrictions and voluntary codes with limited success.

Germany imposed a ban on the use of commercial sunbeds by those aged under 18 years in 2009, and issued a set of UV protection regulations in 2012. Such a ban also exists in Austria, Belgium, France, Iceland, Ireland, Israel, Italy, Norway, Portugal and Spain. 

Tanning salons in Germany must limit UV irradiation to a low level, sunbeds must have timers so that they automatically switch off, and goggles must be provided to protect users’ eyes. In addition, staff must advise customers with skin that burns, but does not tan, against using sunbeds.

“A 2012 study shows that, despite the current ban, around 5% of minors continue to use sunbeds,” says Christian Greipl, head of the Radiological Protection Directorate in Germany’s federal environment ministry.

Also, despite the requirement for the presence of trained staff, self-service tanning salons are still in operation, he says, referring to the *Sunbed-use: needs for action study* known as the “SUN-Study”.

According to that study, the typical sunbed user in Germany is female and between 18 and 25 years, and women use sunbeds twice as often as men of the same age.

“Although the health risks of sunbed use are scientifically well documented, many people in Germany did not appear to be aware of them,” Greipl says, adding: “Many sunbed users reported continued use despite knowing about the potential health risks.”

Compliance is a big challenge, he says. Yet, while a general ban may be the only way to ensure comprehensive, effective radiation protection to safeguard human health, such a ban is deemed – according to the government’s assessment so far – an infringement on personal freedoms enshrined in the constitution.

“Because minors are especially vulnerable and still developing, the government’s duty of care towards this group is strong enough to warrant a ban to protect them from the health risks of artificial UV radiation,” Greipl says.

**Figure:**
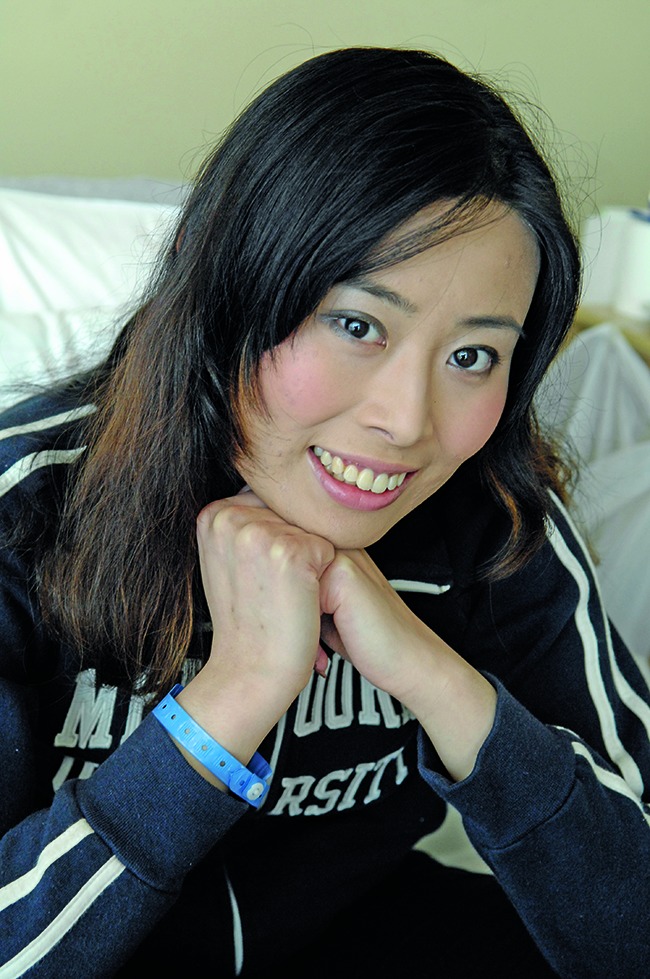
Clare Oliver

Courtesy of the Cancer Council Victoria

**Figure:**
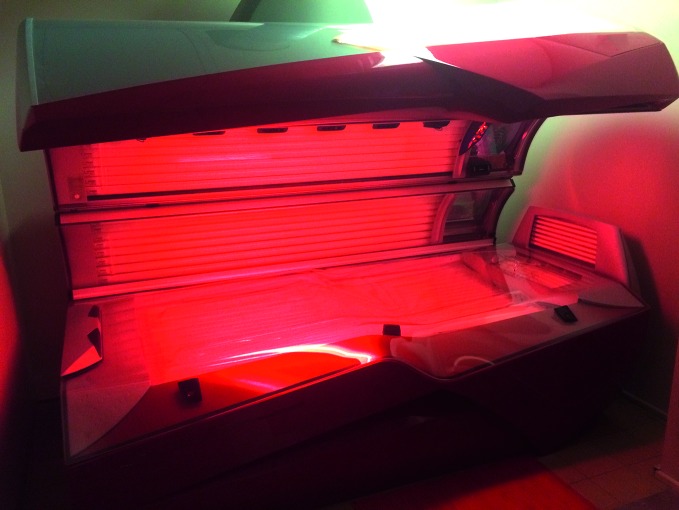
This solarium is unattended and the sunbeds are operated by slot machine, so anyone – even minors – can enter the premises and use them.

WHO/Socheata Son

